# New Insight into Polydopamine@ZIF-8 Nanohybrids: A Zinc-Releasing Container for Potential Anticancer Activity

**DOI:** 10.3390/polym10050476

**Published:** 2018-04-27

**Authors:** Jingyu Ran, Cong Wang, Jinjuan Zhang, Wei Wang, Lihua Xiao, Shaoyi Jia, Ze Wang, Weidang Wu, Jun Xiao, Xinyu Wu

**Affiliations:** 1 School of Chemical Engineering and Technology, Tianjin University, Tianjin 300072, China; wangcongchem@tju.edu.cn (C.W.); jiashaoyi@tju.edu.cn (S.J.); 2 School of Chemical Engineering, Guizhou Institute of Technology, Guiyang 550003, China; wangwei350339930@163.com (W.W.); 20130209@git.edu.cn (L.X.); 3 Basic Medical Experimental Teaching Center, School of Basic Medical Science, Guizhou Medical University, Guiyang 550025, China; zjj216@163.com; 4 The State Key Laboratories of Drug Delivery Technology and Pharmacokinetics, Tianjin Institute of Pharmaceutical Research Co. Ltd., Tianjin 300193, China; wangze9452@163.com; 5 Tianjin University of Traditional Chinese Medicine, Tianjin 300193, China; 6 School of Biology and Engineering, Guizhou Medical University, Guiyang 550025, China; xiaojun@gmc.edu.cn; 7 School of Pharmaceutical Science, Guizhou Medical University, Guiyang 550025, China; xinyuwu1314@163.com

**Keywords:** polydopamine, ZIF-8, Zinc-releasing, anticancer activity

## Abstract

Despite the initial evidence on the role of zinc and zinc transporters in cancer prevention, little attention has been paid to the zinc-derived compounds. In the present work, we reported a strategy to prepare a kind of zinc-releasing container with enhanced biocompatibility and release dynamics using ZIF-8 nanocrystals as the sacrificial templates. Transmission electron microscopy (TEM) analysis demonstrated that the ZIF-8 nanocrystals were gradually etched out in the aqueous media within 48 h, resulting in hollow nanocapsules. Notably, we found the self-polymerization of dopamine can form nanoshells around the ZIF-8 nanocrystals, which served as a type of functional membranes during the release of zinc. More interestingly, PDA@ZIF-8–based nanohybrids expressed stronger inhibition to the cancer cell growth, which implied that the nanohybrids could be a drug carrier for chemotherapy. This study broadens the biomedical application of ZIF-8 and also provides a versatile strategy toward the development of multifunctional delivery system.

## 1. Introduction

It is well known that zinc is an essential micronutrient for mammals [1]. Unlike other metal ions, zinc has a long-standing safety record that has been studied for its chemopreventive effects in several types of cancers [2], such as prostate [3,4], breast [5,6], pancreas [7,8], etc. Fong et al. have shown that zinc supplementation rapidly induced apoptosis in esophageal epithelial cells and may have a role in the prevention of esophageal cancer [9]. Koji Yoshinaga et al. have demonstrated that polaprezinc increased the low zinc concentration in the lingual epithelium and may improve the cellular functions, especially for the proliferation [10]. Despite the initial evidence on the role of zinc and zinc transporters in cancer prevention, however, to the best of our knowledge, little attention has been paid to zinc-derived compounds [2]. Therefore, aiming at the fabrication of zinc-derived compounds for efficient cancer therapy is urgently required.

Zeolitic imidazolate frameworks (ZIFs) are a subclass of metal organic frameworks (MOFs), owning high thermal stability, large surface area, and high porosity. As a representative of ZIFs, ZIF-8 (also known as MAF-4), an active catalyst with large cavities interconnected by narrow windows, exhibits the sodalite (SOD) zeolite-type structure, which is built from lewis acid zinc (II) ions and basic imidazolate ligands [11]. Significant progress has been made regarding biomedical applications of ZIF-8 [12,13,14,15,16], studies also showed that different target molecules could be encapsulated into ZIF-8 before the crystal formation, and several post-synthetic approaches have been developed to endow the external surface of ZIF-8 with more properties [17,18,19,20], but little attention is paid on the zinc included in ZIF-8.

In our previous works, we obtained polydopamine (PDA) nanocapsules by utilizing ZIF-8 nanocrystals as the sacrificial template without any special etchant [21]. PDA is of increasing interest in various fields based on its fascinating properties. As a facile and universal approach, PDA-coated nanoparticles showed negligible cytotoxicity and good biocompatibility, and were stable in vivo for several weeks [22,23]. PDA shell has also been proposed as a new generation of high photothermal conversion agent, aromatic anticancer drug carrier, and molecular imprinted polymer [24,25]. According to our research results, the as-prepared ZIF-8 nanocrystals were unstable under the moist environment. We therefore hypothesized that the zinc would be released from the ZIF-8 when they began to dissolve in the aqueous media. In line of these, herein we selected the aqueous-solution-unstable ZIF-8 nanocrystals as the host materials to investigate our hypothesis.

**Scheme 1:**
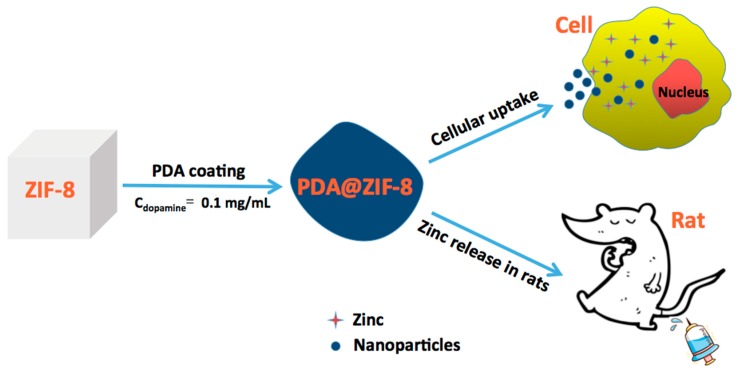
Preparation of PDA@ZIF-8 nanohybrids, in vitro cell model, and in vivo animal model used in this study (not to scale).

In the current contribution, we demonstrate that the ZIF-8 nanocrystals can be used as sacrificial templates for the preparation of delivery system to exert the synergistic effects of zinc and targeted molecules for potential anticancer activity. Scheme 1 is a schematic illustration for the whole experimental process. The simultaneous self-etching and self-polymerization approach presented herein a few of distinct advantages: (1) the ZIF-8 endowed the porous property to PDA shells, which served as a slow-release membrane during the release of zinc; (2) the delivery system can be obtained using a facile preparation process under mild conditions; and (3) the nanohybrids could not only enhance the biocompatibility of ZIF-8 nanocrystals as a drug carrier for chemotherapy but also effectively killed the cancer cells by the synergetic effects of zinc and hydrophobic drug. Overall, given the good biocompatibility and enhanced anticancer properties of nanohybrids, we anticipate their application in a broad set of biomedical applications.

## 2. Experiments

### 2.1. Materials

Dopamine (DA) hydrochloride (Sigma, AP), Zn(NO_3_)_2_·6H_2_O, (98%, Sigma-Aldrich, St. Louis, MO, USA), 2-methylimidazole (2-MeIM, 99%, Aldrich), cetyl trimethyl ammonium bromide (CTAB, 98%, Aldrich), Melphalan (Sigma, 109K1617), Tris (hydroxymethyl) aminomethane, hydrochloric acid, methanol, Rhodamine (Tianjin Chemical Company, AP, Tianjin, China). The colon cancer cell line HCT8 and human embryonic kidney cell line HEK293 obtained from cell bank of China Academy of Science (Shanghai, China), and MCF-7 cells obtained from the China Center for Typical Culture Collection (Wuhan, China) were cultured in DMEM (Gibco, Waltham, MA, USA) supplemented with 10% fetal bovine serum (FBS, Suhr, Switzerland), 2 mg/mL NaHCO_3_, and 100 U/mL penicillin–streptomycin. The cells were incubated at 37 °C in a humidified 5% CO_2_ atmosphere. The zinc assay kit was bought from Nanjing Jiancheng Biotechnology Co. Ltd. (Nanjing, China). The healthy rats and diet were provided by Beijing Vital River Laboratory Animal Technology Co., Ltd. (Beijing, China). The license number was SCXK (Jing) 2012-0001, and the test animal quality certificate number was No. 11400700058460. All chemicals were used without further purification, and Milli-Q water was used throughout this study.

### 2.2. Synthesis of ZIF-8 Nanocrystals

The synthesis of ZIF-8 nanocrystals followed the previous established method with some modifications [26]. In brief, 6.0 mL of 33.6 mM Zn(NO_3_)_2_·6H_2_O aqueous solution was mixed with 100 μL of 100.0 mM CTAB aqueous solution. After stirring for 5 min at room temperature, 10.0 mL of 1.10 M 2-MeIM aqueous solution was added under stirring for another 5 min. The mixture was stirred (400 rpm) at room temperature for 24 h. The samples were collected by centrifugation, washed several times with methanol, and dried.

### 2.3. Fabrication of PDA@ZIF-8 Nanohybrids

DA hydrochloride (10.0 mg) was added to 100.0 mL Tris-HCl buffer (10.0 mM, pH 8.5). Then, 50.0 mg of the as-prepared ZIF-8 nanocrystals was added to the above solution. After being stirred (400 rpm) at room temperature for 1–48 h, the samples were collected by centrifugation, washed several times with methanol and dried.

### 2.4. Preparation of PDA@ZIF-8@Melphalan Nanohybrids

Melphalan (30 mg) was dispersed ultrasonically in 40 mL of 1.10 M 2-MeIM aqueous solution to form solution A. 24.0 mL of 33.6 mM Zn(NO_3_)_2_·6H_2_O aqueous solution was mixed with 400 μL of 100.0 mM CTAB aqueous solution to generate solution B. DA hydrochloride (10.0 mg) was added to 100.0 mL Tris-HCl buffer (10.0 mM, pH 8.5) to generate solution C. Then, solutions A and B were mixed together under magnetic stirring for 24 h. The white precipitates were collected, washed with methanol, and dried for use. After that, 50 mg of as-prepared samples was ultrasonic dispersed in 2 mL methanol for 20 min to generate a suspension, and the suspension was added into solution C under magnetic stirring for 1 h. The cyaneous precipitates were collected, washed with methanol, and dried for use.

### 2.5. General Characterization 

Scanning electron microscopy (SEM) measurements were performed using a FEI Novsa Nano SEM 450 system at an accelerating voltage of 3 kV. Samples were placed on silicon wafer, and then coated with gold. Transmission electron microscope (TEM) measurements were performed using a FEI Tecnai G^2^F20 S-Twinsystem at an accelerating voltage of 200 kV, samples were placed on holey carbon grids. The Brunauer-Emmett-Teller (BET) surface area was determined at 77 K on a Micromeritics ASAP 2020 M instrument. Before analysis, the samples were degassed at 150 °C for 12 h. Thermogravimetric analysis (TGA) was performed using a thermogravimetric analyzer (TA Instruments Q50) (New Castle, DE, USA). The sample was heated from room temperature to 600 °C at a rate of 10 °C/min under nitrogen atmosphere.

### 2.6. Confocal Laser Scanning Microscopy

The cellular internalization of nanoparticles was analyzed using nanoparticles labeled with Rhodamine, MCF-7 cells were used in this study. The seeding of the cells was seeded (1 × 10^5^ cells/well) in 6-well tissue culture plate and cultured at 37 °C under 5% CO_2_/95% air condition. Cells were then incubated with Rhodamine B-labeled PDA@ZIF-8@Melphalan (0.5 mg/mL) for 30 min in CO_2_ incubator, followed by five washes with phosphate-buffered solution (PBS). Samples were examined under a fluorescence laser scanning confocal microscope (FV1000; Olympus, Tokyo, Japan).

### 2.7. Cytotoxicity Assay of ZIF-8 and PDA@ZIF-8

The cytotoxicity study of ZIF-8 nanocrystals and PDA@ZIF-8 nanohybrids was performed with colon cancer cell line HCT8 and normal model cell line HEK293. 1.5 × 10^5^/mL cells were incubated together with 1.5 mg/mL samples in 24 well plates. After 48 h incubation, the culture media were collected, and the cells were washed with (PBS). The cells within 24 well plates were digested with 0.25% (*m*/*v*) trypsin for 5 min and stopped as well as washed with PBS. The digested cells together with the cells in culture media and washing buffer were centrifuged again for 5 min (800 rpm). The cytotoxicity of zinc was determined by flow cytometer reflected by the live cell number (LN), dead cell number (DN) and the rate of the live cell number to dead cell number (RLD). Cell numbers were counted using Countstar IC1000 (Ruiyu Biotech Co., Ltd., Shanghai, China).

### 2.8. The Determination of Zinc Concentration 

The concentration of zinc was determined using assay kits. 15 μL of cell lysis solution or serum samples were mixed with 240 μL of Regent 1 [Ascorbic acid (50 mmol/L) + HEPES buffer (200 mmol/L, pH6.0) + Trisodium citrate dehydrate (0.2 mol/L)], placed in the water bath at 37 °C for 5 min, and the absorbance was tested at λ = 578 nm (*A*_1*n*_); then added 60 μL of Regent 2 [5-Br-PAPS (20 μmol/L)], also placed in the water bath at 37 °C for 5 min and the absorbance was tested at λ = 578 nm again (*A*_2*n*_). As a standard solution, the absorbance of zinc chloride (*C*_0_ = 30.6 μmol/L) was also tested as above (A_10_, A_20_). Zinc concentration was calculated as follow: *C = C*_0_ × [(*A*_2*n*_ *− A*_1*n*_)/(*A*_20_ − *A*_10_)].

### 2.9. In Vitro Zinc Release of ZIF-8 and PDA@ZIF-8

The release dynamics of zinc from ZIF-8 and PDA@ZIF-8 were also carried out in HCT8 and HEK293. The cell lines were revived from liquid nitrogen and cultured continuously for 2–3 generations in DMEM supplemented with 10% FBS. The obtained cells were seeded in 24-well culture plates at a concentration of 1.5 × 10^5^ cells per mL. 1 mg/mL of the nanoparticles were added into the cells in DMEM without FBS (0.5 mL/well), the wells (*n* = 3–5) were washed with PBS and lysed with deionized water (dH_2_O) by multigelation at time 0, 0.5, 1, 2, 4, 6, 8, 12, 24 and 48 h, respectively.

### 2.10. In Vivo Zinc Release of ZIF-8 and PDA@ZIF-8

The release dynamics of zinc from ZIF-8 and PDA@ZIF-8 were evaluated in Sprague Dawley (SD) rats. The twelve specific-pathogen free SD rats weighed 200–240 g were divided into two groups, which were injected intraperitoneally (i.p.) at a dosage of 30 mg/mL/animal. Blood was sampled from eye venous plexus at 0, 0.5, 1, 2, 4, 8, 12, 24, 48, 72, 96, 120 and 168 h, respectively after injection. Animal experiments were performed according to Guidelines for Animal Care and Use Committee, Tianjin Institute of Pharmaceutical Research Co., Ltd. (Tianjin, China), and the license number of the test unit was SYXK (Jin) 2011-0005.

### 2.11. In Vitro Anti-Tumor Activity

The in vitro anti-tumor activity of the nanoparticles was studied by MTT assay using MCF-7 cell line. Briefly, the MCF-7 cells were grown in DMEM supplemented with 10% (*v*/*v*) FBS, 100 IU/mL penicillin G sodium and 100 μg/mL streptomycin sulfate. 100 µL of the cells (8 × 10^5^/mL) were transferred to each well of 96-well plates and incubated overnight at 37 °C in a stove with 5% CO_2_. Subsequently, 100 µL of Melphalan, ZIF-8, PDA@ZIF-8, ZIF-8@Melphalan, and PDA@ZIF-8@Melphalan with different concentrations (31.3, 62.5, 125 and 250 μg/mL) were added to the wells and incubated for 24 h. Next, 10 µL MTT (5 mg/mL) was added to each well and incubated for another 4 h. Finally, the supernatant of the wells was discarded and 100 µL DMSO was added into each well. The absorbance was measured at 490 nm by using a BioRad microplate reader. Untreated cells were used as control with 100% viability and cells without addition of MTT were used as blank to calibrate the spectrophotometer to zero absorbance. The cell growth inhibitory rate (GIR) was calculated using an equation as below: GIR = (absorbance control group − absorbance nanoparticle group)/absorbance control group × 100%

### 2.12. Statistical Analysis

Statistical analyses were performed using SPSS for Windows statistical package (version 22.0). The student’s tests (*t*-test) were used for the statistical analysis of these data. A minimum of three independent experiments (in viability assays, three replicates were analyzed in each experiment) was performed for each experimental condition tested. Experimental data were expressed as mean ± standard error and a *p*-value of 0.05 was considered significant.

## 3. Results and Discussion

### 3.1. SEM and TEM Analysis

**Figure 1:**
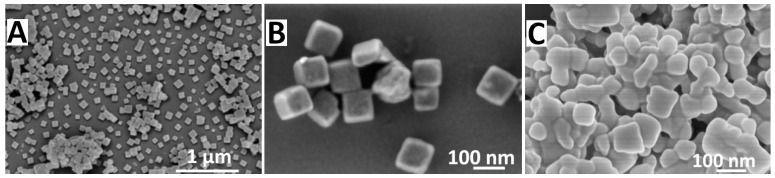
Representative scanning electron microscopy (SEM) images of ZIF-8 (**A**,**B**); and hydrolysis of ZIF-8 in the aqueous media ca. 3 h (**C**).

**Figure 2:**
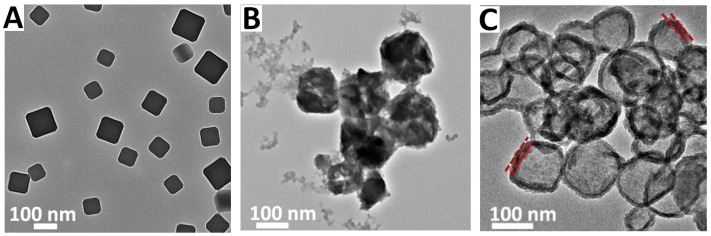
Representative transmission electron microscopy (TEM) images of ZIF-8 and PDA@ZIF-8. (**A**) Pristine ZIF-8 truncated cubes; (**B**) after PDA coating, ca. 3 h; (**C**) immersed in aqueous media, ca. 48 h.

ZIF-8 nanocrystals exhibited a typical truncated cubes shape (Figure 1A,B), but they were not stable in the aqueous media and were etched after 3 h (Figure 1C). TEM images further indicated that the PDA@ZIF-8 nanohybrids were gradually etched out during immersion in the aqueous within 48 h, resulting in hollow nanocapsules, and the PDA nanoshells could be formed on the ZIF-8 nanocrystals by simply dispersing them in the DA hydrochloride solution (Figure 2A–C). In our previous work, we have confirmed that the shell of the nanocapsules is indeed PDA [21]. The same coating procedure was used in the current work to fabricate PDA@ZIF-8 nanohybrids. Based on this fact, we suppose that the zinc would be released when the nanostructure undergoing hydrolysis in the aqueous media. This suggested to us that the zinc of the nanohybrids would be released after getting into the cancer cells. In addition, we observed that the PDA coating exhibited porous as the PDA coating deposited onto the surface of ZIF-8, which owns high porosity. The permanent porosity of the resulting nanoshells was confirmed by nitrogen absorption analysis at 77 K (Table 1). In other words, the ZIF-8 endowed the porous property to PDA coating, but the reduction in the surface area was associated with the incorporation of the PDA coating. The surface areas of PDA@ZIF-8 nanocomposites exceeded 1000 m^2^ g^−1^, such high surface areas could lead to high drug adsorption and release capability.

### 3.2. In Vitro Cytotoxicity Evaluation

**Figure 3:**
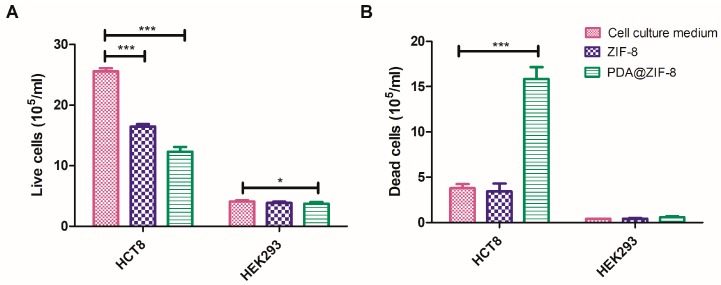
In vitro cytotoxicity test results (* *p* < 0.05, ** *p* < 0.01, *** *p* < 0.001).

The cytotoxicity of ZIF-8 and PDA@ZIF-8 was tested in the cell lines HCT8 and HEK293 (as shown in Figure 3), the cells were incubated with 0.5 mg/mL of ZIF-8, or PDA@ZIF-8. After 48 h, the cell viability was determined by flow cytometer. Cells incubated in culture medium were used as a control group. Previous work has demonstrated that the intracellular total zinc concentration is about 200–500 uM in the most of mammalian cells [9]. Until now, many works have concluded that zinc was relatively harmless compared to several other metal ions with similar chemical properties; only exposure to high doses had a significant correlation with cell viability [27,28]. The molarity of zinc detected in this study was within the allowable concentration range, so we suppose that there is no toxicity effect on the normal cells in theory. As expected, the ZIF-8 and PDA@ZIF-8 both had a very low toxicity for normal cells, but they both exhibited the inhibitory effect on HCT8 cells. Compared with the ZIF-8, PDA@ZIF-8 expressed stronger inhibition to the cancer cell growth. The difference in toxicity could be due to differences in uptake as the phenolic content on PDA makes it more likely to associate with cells [29]. Besides, this may be due to the increased cell permeability of the tumor cells, which promoted the adsorption of zinc, then accelerating the cell apoptosis. This suggested that the nanohybrids could not only enhance the biocompatibility of ZIF-8 as a drug carrier for chemotherapy but also effectively killed the cancer cells.

### 3.3. Zinc Release Investigation in HCT8 and HEK293 

**Figure 4:**
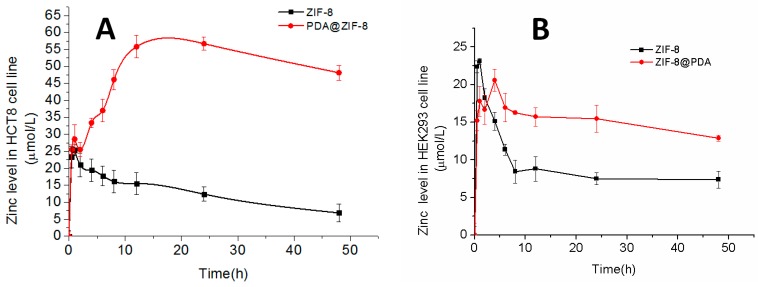
(**A**) The concentration of zinc in HCT8 cell line at different time after incubation with ZIF-8 (black line) and PDA@ZIF-8 (red line); (**B**) The concentration of zinc in HEK293 cell line at different time after incubation with ZIF-8 (black line) and PDA@ZIF-8 (red line). Results are presented as means standard deviation (s.d.) (*n* = 3).

The release dynamics of zinc from ZIF-8 and PDA@ZIF-8 were investigated in vitro. The cell lines HCT8 and HEK293 were incubated with ZIF-8 and PDA@ZIF-8 for 48 h, respectively. As shown in Figure 4A, in HCT8 cell line, the zinc released from PDA@ZIF-8 and reached the maximum concentration (C_max_) (56.74 μmol/L), and after that this concentration level lasted for 12 h. The zinc released from ZIF-8 and the concentration decreased after 1 h when reaching the C_max_ (25.41 μmol/L). In the HEK293 cell line, the zinc released from PDA@ZIF-8 reached the C_max_ (20.56 μmol/L) at 6 h after dosing, but the zinc released from ZIF-8 reached the C_max_ (23.10 μmol/L) at 1 h and decreased drastically (Figure 4B). Although two samples both reached the C_max_ in a short time, the zinc released from PDA@ZIF-8 maintained a relative high concentration within 48 h compared with ZIF-8 group. The phenomenon might be due to the fact that the ZIF-8 were unstable in cell lines, and the zinc was more easily released from only the primitive nanocrystals. In the case of PDA@ZIF-8, the PDA nanoshells slowed the release of zinc, but it inherited the porous property of ZIF-8 nanocrystals, which served as a type of functional membranes during the release of zinc, thereby maintaining a higher concentration over a longer period of time.

### 3.4. Zinc Release Investigation in Rats

**Figure 5:**
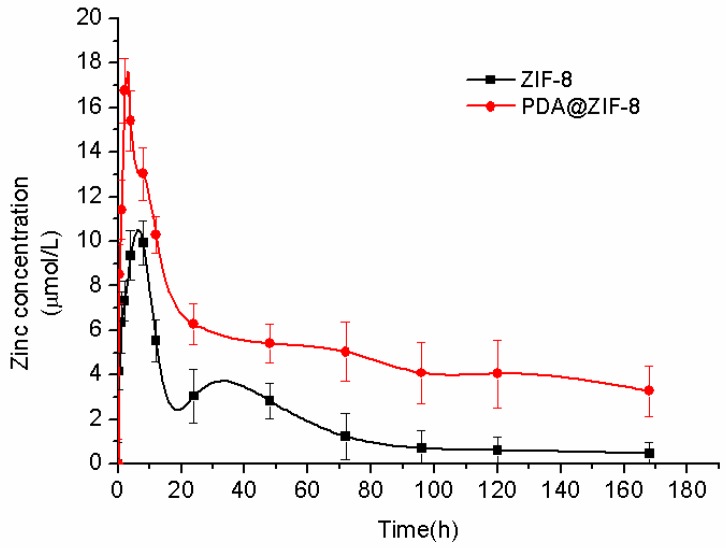
The concentration of zinc in blood at different time after administration with ZIF-8 (black line) and PDA@ZIF-8 (red line). Results are presented as means standard deviation (s.d.) (*n* = 3).

The release dynamics for ZIF-8 and PDA@ZIF-8 in vivo were shown in Table 2 and Figure 5. The area under curve (AUC) of the PDA@ZIF-8 was higher than that of the ZIF-8, suggesting that the zinc within PDA nanoshells were more suitable for cell uptake. The C_max_ of the zinc from PDA@ZIF-8 was also enhanced by 1.64 folds compared with the ZIF-8, and the time (T_max_) reached the peak of the zinc from PDA@ZIF-8 was shorter than the ZIF-8, which suggested that the PDA@ZIF-8 were more feasibly for absorption than the ZIF-8, and the sustained release effect was enhanced. The mean retention time (MRT) of zinc in blood from PDA@ZIF-8 was longer than that of the ZIF-8. In addition, the elimination time (T_1/2_) of zinc from the PDA@ZIF-8 was significantly prolonged compared with the ZIF-8. Such results demonstrated that PDA@ZIF-8 presented improved dynamics properties in vivo, which were due to the formed PDA nanoshells that could serve as molecularly imprinted polymer coatings for protein recognition [25].

### 3.5. Anti-Tumoral Activity for MCF-7 Cells

**Figure 6:**
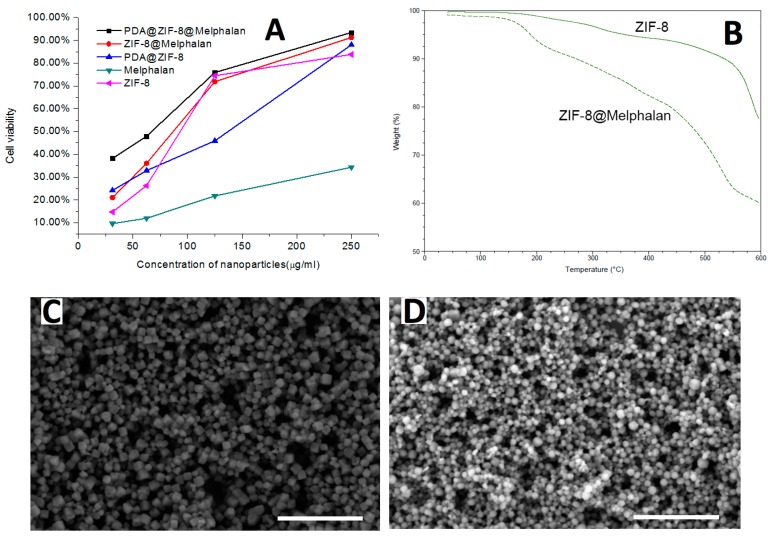
MTT assays of in vitro cytotoxic activity of Melphalan, ZIF-8@Melphalan, PDA@ZIF-8, PDA@ZIF-8@Melphalan and ZIF-8 on MCF-7 cells (**A**); TGA of ZIF-8 and ZIF-8@Melphalan (**B**); representative SEM images of PDA@ZIF-8 (**C**) and PDA@ZIF-8@Melphalan (**D**). All scale bars are 1 μm.

The in vitro cytotoxic activity of Melphalan, ZIF-8@Melphalan, PDA@ZIF-8, PDA@ZIF-8@Melphalan and ZIF-8 was evaluated by the MTT assay using the MCF-7 cell line (Figure 6A). It has been observed that, after encapsulated with Melphalan, most of the nanoparticles retained the truncated cubes shape as primitive ZIF-8, but these changed into almost spherical shape after coated with PDA (Figure 6C,D). The loading percentage of Melphalan was about 7 wt % determined by thermal gravimetric analyses (TGA) (Figure 6B), confirming that the target molecules could be encapsulated into ZIF-8. As demonstrated in Figure 6A, ZIF-8 based nanoparticles totally inhibited cell viability at 250 μg/mL, while a 40% decrease in cell viability was achieved at lower concentrations of PDA@ZIF-8@Melphalan. The cell inhibitory efficiencies of ZIF-8@Melphalan, PDA@ZIF-8, PDA@ZIF-8@Melphalan, and ZIF-8 are higher than that of free Melphalan. Lower cytotoxic activity was observed for Melphalan from 31.25 μg/mL to 250 μg/mL. This might be attributed to the melphalan that is an effective treatment option for patients with multiple myeloma but not for the breast cancer. ZIF-8 exhibited apparent cytotoxicity in MCF-7 without drug loading. This indicated that the extracellular zinc exerts rapid inhibitory effects on cancer cells, for the decomposition of ZIF-8 generates massive zinc in cells. More importantly, the cytotoxicity was enhanced by the combination of ZIF-8 and Melphalan, and the synergistic effect was obvious in the inhibition of cell growth. These results are in accordance with those of anti-tumoral activity studies: zinc can significantly suppress cell proliferation in cell line, extracellular zinc alone was sufficient to induce metabolic changes or even apoptosis in cancer cell lines [30,31,32], and zinc can affect the functions of a number of ion channels and transporters of cells [33].

### 3.6. In Vitro Cellular Uptake of PDA@ZIF-8@Melphalan

**Figure 7:**
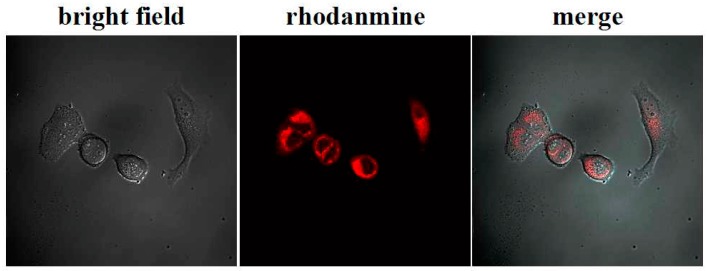
Representative fluorescence microscopy images of MCF-7 cells were treated with PDA@ZIF-8@Melphalan. Nanoparticles are labelled with Rhodanmine.

To check if the PDA@ZIF-8@Melphalan nanohybrids were internalized in tumor cells, cellular uptake was observed using confocal microscopy. Figure 7 showed that at high resolution the engulfment of the nanoparticles by MCF-7 cells, and the nanoparticles incubated in cell culture medium demonstrated high stability. In addition, no free dye was detectable after 30 min of incubation in cell culture medium. It suggested that the existence of PDA allows their effective incorporation into cellular membranes, and the well-described ability of PDA to bind with cell membrane can be responsible for the efficient internalization of PDA@ZIF-8@Melphalan nanohybrids observed. The CLSM results are in accordance with the cell inhibition results of the MTT assay.

## 4. Conclusions

In summary, the main finding herein is that the aqueous solution unstable ZIF-8 nanocrystals can serve as a sacrificial carrier for zinc-releasing in chemotherapy. The detailed information of zinc release in vitro and in vivo suggests that the PDA coating may be exploited for drug delivery system to achieve constant drug concentration over prolonged periods, and the PDA@ZIF-8 nanohybrids showed improved dynamic properties than that of the ZIF-8 nanocrystals. We envision that this novel set of nanohybrids will allow the design and fabrication of multifunctional nanomedical platforms for biomedical application including targeted delivery, controlled release, and drug encapsulation.

## Figures and Tables

**Table 1:** ZIF-8 and PDA@ZIF-8 physicochemical properties.

Sample	Average Particle Size ^‡^ (nm)	BET Surface Area (m^2^ g^−1^)	Pore Volume (cc g^−^)
ZIF-8	90 ± 15	1278	1.24
^†^ PDA@ZIF-8	100 ± 12	1044	0.40

^†^ The coating condition is as follows: C_dopamine_ = 0.1 mg mL^−1^ and the coating time is 1 h; ^‡^ The values were calculated from SEM images by Nano Measure software.

**Table 2:** Zinc release dynamic parameters in vivo ^†^.

Parameter	Unit	ZIF-8	PDA@ZIF-8
AUC	μmol × h/L	339.44 ± 66.37	730.29 ± 93.13
C_max_	μmol/L	10.43 ± 2.77	17.17 ± 2.23
T_max_	h	8.20 ± 1.00	3.67 ± 0.58
MRT	h	47.80 ± 8.21	64.99 ± 3.24
T_1/2_	h	78.89 ± 38.13	173.05 ± 21.92

^†^ PDA@ZIF-8 vs. ZIF-8, *p* < 0.05, *t*-test, *n* = 6.
